# Dissecting Kawasaki disease: a state-of-the-art review

**DOI:** 10.1007/s00431-017-2937-5

**Published:** 2017-06-27

**Authors:** S. M. Dietz, D. van Stijn, D. Burgner, M. Levin, I. M. Kuipers, B. A. Hutten, T. W. Kuijpers

**Affiliations:** 1 Department of Pediatric Hematology, Immunology and Infectious Diseases, Emma Childrens Hospital, Academic Medical Centre (AMC), Meibergdreef 9, 1105 AZ Amsterdam, The Netherlands; 2 Murdoch Childrens Research Institute, Parkville, VIC Australia; 3 Department of Pediatrics, Melbourne University, Parkville, VIC Australia; 4 Department of Pediatrics, Monash University, Clayton, VIC Australia; 5 Pediatric Infectious Diseases Group, Division of Medicine, Imperial College London, London, UK; 6 Department of Pediatric Cardiology, Emma Childrens Hospital, Academic Medical Centre (AMC), Amsterdam, The Netherlands; 7 Department of Clinical Epidemiology, Bioinformatics and Biostatistics, AMC, Amsterdam, The Netherlands

**Keywords:** Kawasaki disease, Coronary artery aneurysms, Intravenous immunoglobulins, Genetics

## Abstract

Kawasaki disease (KD) is a pediatric vasculitis with coronary artery aneurysms (CAA) as its main complication. The diagnosis is based on the presence of persistent fever and clinical features including exanthema, lymphadenopathy, conjunctival injection, and changes to the mucosae and extremities. Although the etiology remains unknown, the current consensus is that it is likely caused by an (infectious) trigger initiating an abnormal immune response in genetically predisposed children. Treatment consists of high dose intravenous immunoglobulin (IVIG) and is directed at preventing the development of CAA. Unfortunately, 10–20% of all patients fail to respond to IVIG and these children need additional anti-inflammatory treatment. Coronary artery lesions are diagnosed by echocardiography in the acute and subacute phases. Both absolute arterial diameters and *z*-scores, adjusted for height and weight, are used as criteria for CAA. Close monitoring of CAA is important as ischemic symptoms or myocardial infarction due to thrombosis or stenosis can occur. These complications are most likely to arise in the largest, so-called giant CAA. Apart from the presence of CAA, it is unclear whether KD causes an increased cardiovascular risk due to the vasculitis itself.

*Conclusion*: Many aspects of KD remain unknown, although there is growing knowledge on the etiology, treatment, and development and classification of CAA. Since children with previous KD are entering adulthood, long-term follow-up is increasingly important.

**Table:** 

**What is known:** *• Kawasaki disease (KD) is a pediatric vasculitis with coronary artery damage as its main complication*. *• Although KD approaches its 50th birthday since its first description, many aspects of the disease remain poorly understood*.
**What is new:** *• In recent years, multiple genetic candidate pathways involved in KD have been identified, with recently promising information about the ITPKC pathway*. *• As increasing numbers of KD patients are reaching adulthood, increasing information is available about the long-term consequences of coronary artery damage and broader cardiovascular risk*.

## Introduction

Mucocutaneous lymph node syndrome is an acute vasculitis first described by Dr. Tomisaku Kawasaki in 1967 [60]. This condition, now known as “Kawasaki disease (KD),” is increasingly recognized in Western countries but has a greatly increased incidence in Japan and Asia. The main complication is coronary artery damage or coronary artery aneurysms (CAA) and KD is the leading cause of pediatric acquired heart disease in asset-rich countries.

## Epidemiology

Kawasaki disease is commonest in infants and young children. The incidence varies markedly between ethnic groups. The incidence of KD in recent European studies is 5–10 per 100,000 children under the age of 5 years [45, 55, 105, 120]. A considerably higher incidence is reported in Asian countries [14, 44]. The highest incidence is found in Japan; the most recent nationwide survey reported an incidence of 265/100,000 children under the age of 5 in 2012, and suggested that the incidence of KD is still rising [78]. Most patients are 6 months to 5 years old, although cases in older children and adults also occur [36]. The male/female ratio is approximately 1.5 to 1 [120].

## Diagnosis

The diagnosis of KD is based on the presence of clinical features of persistent fever in combination with a polymorphous exanthema, cervical lymphadenopathy, non-purulent conjunctival injection, changes of the lips and oral cavity (including strawberry tongue, cracked lips, redness of the mucosae), and changes in extremities (swelling and redness of the palms, desquamation in the subacute phase) [83]. In the most recent American Heart Association (AHA) guidelines, persistent fever is classified as ≥5 days, but in the presence of four or more symptoms, the diagnosis can be made with only 4 days of fever [83, 99]. “Complete” KD is defined as fever and ≥4 out of the 5 symptoms. It is important to appreciate that criteria may present successively instead of simultaneously. The AHA has created an algorithm to increase the possibility of “incomplete” KD in case ≤3 criteria are present. This algorithm includes CA abnormalities on echocardiography and/or laboratory abnormalities [83]. There is no diagnostic test for KD, and the diagnosis may be delayed or overlooked. To improve diagnosis, multiple new biomarkers have been studied, but none has so far proved specific for KD [96]. Classification tools have been developed to aid in the differentiation between KD and other febrile illness, although the utility as a point-of-care diagnostic test remains unproven [46, 74].

## Etiopathogenesis

Although the etiology of KD is unknown, the current consensus is that it is likely caused by an (infectious) trigger initiating an abnormal immune response in genetically predisposed children. An infectious etiology is suspected due to the symptomatology of KD resembling common childhood infections, once-in-a-lifetime occurrence at young age (although second manifestations do occur), spatial and temporal clustering, and the clear seasonal pattern in high-incidence countries [57, 78].

### Infectious agents

Multiple viral infectious triggers have been suggested, including coxsackie virus, parainfluenzavirus, respiratory syncytial virus, human metapneumovirus, chikungunya, and cytomegalovirus. In fact, two recent studies showed that up to about half of all KD patients had one of more respiratory viruses detected by PCR, but their etiological role is unproven [13, 128]. Also, the possibility of a respiratory RNA virus has been suggested by ultrastructural studies of autopsy specimens [102, 103]. However, no virus has repeatedly been confirmed in KD studies.

Bacteria have also been suggested as the trigger of KD, with research mainly focusing on bacterial superantigens. Superantigens produced by several bacteria are able to stimulate a large percentage of T cells by binding to the Vβ region of T cell receptors and so stimulate the production of pro-inflammatory cytokines. One study looking at five superantigens (streptococcal pyrogenic exotoxin (SPE)-A, C, G, and J, and toxic shock syndrome toxin-1 (TSST-1)), found these in 70% of stool samples collected from acute KD patients as opposed to 27% in healthy controls visiting the same center for vaccinations [117]. Another study found significantly increased IgM antibodies against five superantigens (staphylococcal enterotoxin A, B, and C, TSST-1, and SPE-A) [80]. Nevertheless, the role of superantigens in KD remains unclear.

### Immunological response

The encounter of a susceptible individual with the unknown agent probably leads to an (exaggerated) immune response involving innate and adaptive pathways. Multiple studies have been performed, both evaluating animal models and immune response in the peripheral blood as well as immune infiltration in the coronary arteries [52, 131].

The general paradigm of the immune response is an imbalance between pro-inflammatory and anti-inflammatory pathways. For example, regulatory T cells, a subset of T cells limiting inflammation, have been shown to be important in the vascular inflammation [37]. Also, the IL-1 signaling pathway is upregulated, with upregulated IL-1 pathway genes and increased IL-1 concentrations in peripheral blood of KD patients during the acute phase [50, 119]. Recently, it has become clear that inflammasomes, multiproteins that are part of the innate immune system, are induced by the *NLRP3* gene and promote the production of IL-1β and IL-18, play a role in KD [2].

In the coronary arteries, immune infiltration of the arterial wall with neutrophils, CD8+ cytotoxic T cells, Ig-A producing plasma cells, and macrophages have been found, accompanied by pro-inflammatory cytokines which may vary in proportion and contribution over time [6].

## Genetics

Genetics are considered to contribute to susceptibility to KD, and probably to CAA and response to treatment [91, 132]. A number of genome-wide association studies (GWAS) have been performed [7, 63, 69, 72, 92, 94, 126]. Apart from the GWAS, multiple studies have identified specific single nucleotide polymorphisms (SNPs) in several genes. Most of these candidate genes have an immune regulatory function. Table 1 shows some of the key pathways and SNPs associated with KD susceptibility, CAA development, and intravenous immunoglobulin (IVIG) resistance.

**Table 1:** Candidate genes and pathways associated with disease susceptibility, CAA development, and IVIG resistance

Candidate pathway	Ethnicity	Reference	Included cases/controls	Susceptibility to KD (gene)	Susceptibility to CAA (gene)	Susceptibility to IVIG resistance (gene)
*ABCC4*	European descent (case-control), Australia, NL, USA, UK (family based)	Khor et al. [62]	26 pedigrees, case-control = 119/225, family based = 1093 cases, 1621 parents, 198 siblings	Yes (*ABCC4*)	x	x
*ANGPT*	Australia, US, UK (family based) and Dutch Caucasian (case-control)	Breunis et al. [5]	462 complete trios, case control: 123/171	Yes (*ANGPT1*)	Yes (*ANGPT2*)	x
Intergenic region *BLK-FAM167A*	Japanese	Onouchi et al. [94] (GWAS)	428/3379	Yes (*FAM167A-BLK*)	x	x
		Onouchi et al. [94] (Replication 1)	470/378	“	x	x
		Onouchi et al. [94] (Replication 2)	284/569		x	x
	(Han) Chinese	Yan et al. [130]	358/815	Yes (*FAM167A-BLK*)	No	x
	Taiwanese	Lee et al. [72] (GWAS)	622/1107^a^	Yes (*BLK*)	x	x
		Lee et al. [72] (replication)	261/550	“	x	x
		Lou et al. [77]	428/493	Nominal association (*BLK*)	x	
	Korean	Chang et al. [12] (GWAS)	186/600^b^	Yes (*BLK*)	x	x
		Chang et al. [12] (Replication)	288/498	“	x	x
	US (European, Asian, Hispanic, Mixed, African American, Native American, Samoan).	Onouchi et al. [94]	503 trios	Yes (*FAM167A-BLK*)	x	x
	European	Chang et al. [12]	405/6252	Yes (*BLK*)	x	x
*CD40*	Japanese	Onouchi et al. [93]	427/476	No *(CD40L*)	Yes (*CD40L*)^c^	x
		Onouchi et al. [94] (GWAS)	428/3379^d^	Yes (*CD40*)	x	x
		Onouchi et al. [94] (replication 1)	470/378	“	x	x
		Onouchi et al. [94] (replication 2)	284/569	“	x	x
	Han Chinese	Kuo et al. [67]	428/493	Yes (*CD40*)	Yes (*CD40*)^d^	x
		Lou et al. [77]	381/569	Nominal association (*CD40*)	x	x
	Taiwanese	Lee et al. [72] (GWAS)	622/1107^a^	Yes (*CD40*)	x	x
		Lee et al. [72] (replication)	261/550	“	x	x
	European	Shendre et al. [108]	112 complete trios	Yes (*CD40*)	x	x
*FCGR2/3*	Japanese	Taniuchi et al. [123]	65/566	Yes (*FCGR3a*)	Yes (*FCGR2a*)	x
		Onouchi et al. [94] (GWAS)	428/3379^e^	Yes (*FCGR2a*)	x	x
		Onouchi et al. [94] (replication 1)	470/378	“	x	x
		Onouchi et al. [94] (replication 2)	284/569	“	x	x
	(Han) Chinese	Duan et al. [32]	428/493	Yes (*FCGR2A*)	No	x
		Yan et al. [130]	358/815	Yes (*FCGR2A*)	No	No
		Khor et al. [61]	130/568	Yes (*FCGR2A*)	x	x
	Taiwanese	Khor et al. [61]	438/446	Yes (*FCGR2A*)	x	x
	Caucasian	Shrestha et al. [111]	176/369	x	x	Yes (*FCGR2B*)
		Shrestha et al. [110]	156 trios, 75 single parent-child	Yes (*FCGR2A*)	Yes (*FCGR2a/3B*)^f^	Yes (*FCGR3B*)
	European	Khor et al. [61] (GWAS)	405/6252^g^	Yes (*FCGR2A*)	x	x
ITPKC	Japanese	Onouchi et al. [92] (Indep. set 1)	94^h^	Yes (*ITPKC*)	Yes (*ITPKC*)	x
		Onouchi et al. [92] (Indep. set 2)	276/282	“	“	x
		Onouchi et al. [92] (Indep. set 3)	267/752	“	“	x
		Onouchi et al. [95]	546/947	Yes (*ITPKC*)	Yes (*ITPKC*)^i^	Yes (*ITPKC*)^i^
	Chinese	Peng et al. [97]	223/318	Yes (*ITPKC*)	Yes (ITPKC)	No
		Khor et al. [61]	130/568	Yes (*ITPKC*)	x	x
	Taiwanese	Chi et al. [18]	385 (of which 158 trios)/1158	No	No	x
		Lin et al. [73]	280/492	Yes (*ITPKC*)	No	x
		Kuo et al. [71]	341/1190	Yes (*ITPKC*)	Yes (*ITPKC*)^j^	No
		Above-mentioned studies combined	999/2781	Yes (*ITPKC*)	x	x
		Kuo et al. [68]	381/569	No^k^	Yes (*ITPKC*)	x
		Khor et al. [61]	438/446	Yes (*ITPKC*)	x	x
	European	Khor et al. [61] (GWAS)	405/6252 (10)	Yes (*ITPKC*)	x	x
		Khor et al. [61] *(Replication)*	605 trios, 139 siblings^l^	“	x	x
	US	Onouchi et al. [92]	209 trios	Yes (*ITPKC*)	Yes (*ITPKC*)	Yes (*ITPKC*)
TGF-β pathway	Japanese	Cho et al. [20]	105/303	Yes (*SMAD5*)	No	x
	Han Chinese	Peng et al. [98]	392/421	Yes (*TGFB2*, *SMAD3*, *ADAM17*)	Yes (*TGFB2*)	No
	Taiwanese	Kuo et al. [70]	381/569	Yes (*SMAD3*)	No	No
	Korean	Choi et al. [21]	105/500	Yes (*TGFBR2*)	Yes (*TGFBR2*)	x
	European descent	Shimizu et al. [109] (cohort 1)	128/159	Yes	x	x
		Shimizu et al. [109] (cohort 2)	451 trios	(*TGFB2*, *TGFBR2*, *SMAD3*)^m^	x	x
		Shimizu et al. [109] (cohort 3)	435	x	Yes^n^	x
		Shimizu et al. [109] (cohort 4)	237	x	Yes^n^	Yes (*TGFB2*, *TGFBR2*, *SMAD 3*, *SMAD5*)
*VEGF*	Japanese	Kariyazono et al. [58]	103/144	x	Yes (*VEGF*, *KDR*)	x
	Taiwanese	Hsueh et al. [51]	93/96	Yes (*VEGF*)	x	x
	Australia, US, UK (family based), Dutch Caucasian (case-control)	Breunis et al. [5]	462 complete trios, case control: 123/171	Yes (*VEGFA*)	Yes (*VEGFR2*)	x
	Dutch Caucasian	Breunis et al. [4]	170/300	Yes (*VEGF*)	No	x

*ABCC4* ATP-binding cassette, subfamily C, member 4, *ANGPT* angiopoetin, *CAA* coronary artery aneurysm, *FCGR* Fc gamma receptor, *GWAS* genome-wide association study, *ITPKC* inositol-triphosphate kinase C, *IVIG* intravenous immunoglobulin, *KD* Kawasaki disease, *NL* Netherlands, *SNP* single nucleotide polymorphism, *TGF-β* transforming growth factor beta, *UK* United Kingdom, *US* United States, *VEGF* vascular endothelial growth factor

^a^Numbers after quality control, starting numbers: 627/1118

^b^Numbers after quality control, starting numbers: 222/600

^c^Significant difference between male patients with CAA (*n* = 58) compared with male patients without CAA (*n* = 195) and with controls, not in female patients

^d^rs4810485 has protective effects for CAL formation in KD patients

^e^Numbers after quality control, starting numbers: 447/3397

^f^Only significant taken in all ethnic groups (White and Asian combined)

^g^Numbers after quality control, starting numbers: 576/7464 cases/controls

^h^ITPKC SNP not genotyped in 564 controls of first cohort

^i^A two-locus model in combination with a SNP in CASP3 was also significantly association with both CAA development and IVIG resistance

^j^CAA of >4 mm or in children over 5 years of age, diameter of at least 1.5 times adjacent segment

^k^Two SNPs found but significance disappeared after correction for multiple testing

^l^Total included trios in study = 740 which 135 of non-European descent, combined analyses

^m^Only one SNP remained significant after Bonferroni correction

^n^Different SNPs found in different cohorts but many of the SNPs colocalized to the first intron of each of the three genes (*TGFB2*, *TGFBR2*, and *SMAD3*)

In the first GWAS that resulted in a significant correlation with susceptibility to KD, we identified the major activating IgG receptor FcgRIIa (CD32a) on immune cells and platelets, encoded by the *FCGR2A* gene at the *FCGR2/3* gene cluster at chromosome 1q23 [7]. Following this study, Japanese and Taiwanese studies also confirmed this genetic association while at the same time characterizing *CD40* and *BLK*, respectively, as being associated with KD, as confirmed for *BLK* in Caucasian KD patients in a subsequent meta-analysis [12, 72, 94]. *CD40* is a protein expressed on antigen-presenting cells, such as dendritic cells, macrophages, and B cells, and interacts with CD40L which is primarily expressed by activated T cells and platelets [49]. The function of *FAM167A* and *BLK* gene is yet to be investigated. The *BLK* gene encodes for tyrosine kinase, which is presumed to play a role in B cell signal transduction [100].

**Fig. 1:**
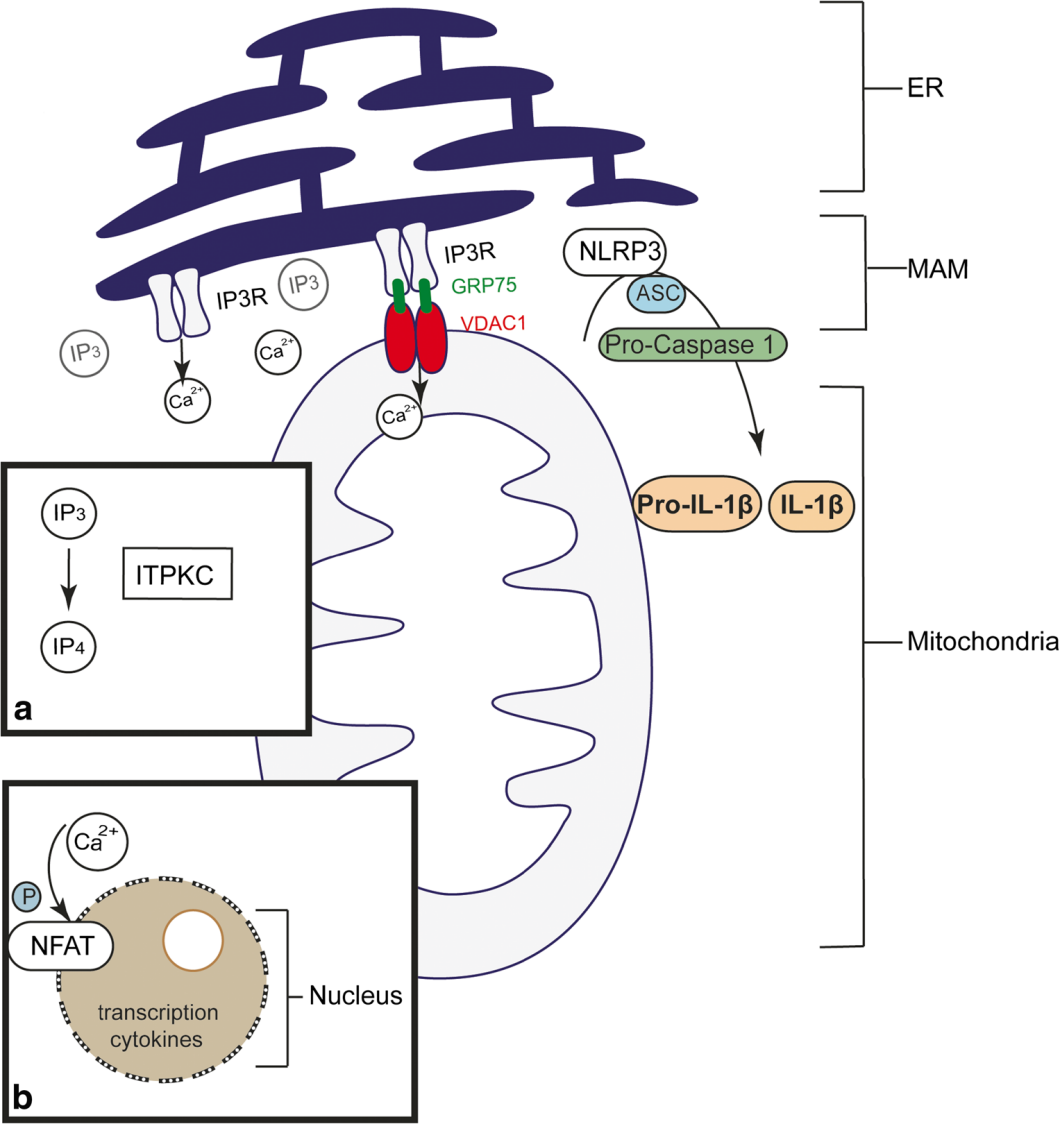
The role of IP3 and ITPKC in calcium signaling. (*a*) ITPKC phosphorylates IP3 to IP4 and modulates the abundance of IP3 and influences the calcium signaling. (*b*) Nuclear factor of activated T cells (*NFAT*) are regulated by calcium signaling and enter the nucleus when dephosphorylated, there it activates cytokine transcription namely IL-2, IFNγ in T cells and Pro-IL1, IL-10, Pro-IL18 in macrophages. *Footnote*: Inositol triphosphate receptor (*IP3R*) forms a bridge between the endoplasmatic reticulum (*ER*) and mitochondria creating a site of contact between the ER and mitochondria called the mitochondria-associated ER membrane (*MAM*). NLRP3 is an inflammasome that forms at or close to the MAM upon cellular activation and ER stress and plays a pivotal role (by activating caspase-1) in the cleavage of pro-IL1b into IL1b and its subsequent secretion. The ER releases calcium into the cytosol and into mitochondria through (a.o.) the IP3R, which is a calcium channel, to which IP3 as an agonist binds to induce calcium release. IP3R binds via glucose-regulated protein 75 (*GRP75*) with the mitochondrial voltage-dependent anion channel 1 (*VDAC1*) which may cause mitochondrial stress and leakage off reactive oxygen species (ROS), both important for inflammasome activation. Macrophages activate via their Toll-like receptors (TLRs) or G-protein coupled receptors several signaling pathways, that result in IP3 formation, NF-kB activation, and/or ER stress.

From alternative genetic studies (non-GWAS), other pathways were found to be involved, such as vascular endothelial growth factor (VEGF) and angiopoietin (ANGPT). ANGPT1 and angiopoietin receptor (TIE-2) promote cell survival and induce anti-inflammatory signals in contrast to ANGPT2 and TIE-2, which have a pro-inflammatory effect with VEGF acting as a co-factor. Also the transcription growth factor beta (TGF-β) pathway may play an important role. TGF-β is important in T cell activation and cardiovascular remodeling. One of the more recent and promising pathways involves the inositol-triphosphate 3-kinase (*ITPKC*) gene. ITPKC expression is part of a transmembrane signaling pathway with release of Ca^2+^ from intracellular storage [48]. Initially, nuclear factor of activated T cells (NFAT) was suggested to be involved in regulating the immune response in KD. The NFAT pathway is calcium-dependent, and when these cytosolic proteins become dephosphorylated, they translocate from the cytoplasm to the nucleus to initiate transcription of downstream target genes including for cytokines such as IL-2, IL-10, and IFNγ. Stimulation of T lymphocytes accommodates the release of inositol-triphosphate (IP3), which increases intracellular Ca^2+^ through the endoplasmic reticulum (Fig. 1). *ITPKC* serves as a negative regulator of the Ca^2+^/NFAT pathway and—at the same time—is also believed to act as a key second messenger in T cell receptor signaling. This would make *ITPKC* responsible for a greater and more prolonged expansion of inflammation, thus creating an increased risk of KD and/or leading to disease severity [76]. Alphonse et al. suggested that the role of *ITPKC* is not T cell-mediated but more monocyte/macrophage-dependent in its impact [2]. They showed that *ITPKC* influences *NLRP3* activation through intracellular calcium levels leading to an increased IL-1β and IL-18 production. Khor et al. performed a global meta-analysis of SNP rs28493229 in *ITPKC* of all performed studies, including GWAS data, showing strong evidence for association with KD (*p* = 8.28 × 10^−22^) [61].

Other pathways and candidate genes have, sometimes inconsistently, been implicated including ATP-binding cassette, subfamily C, member 4 (*ABCC4*), interleukin-4, interleukin-10 and interleukin-18, chemokine receptors, tumor necrosis factor-α and, even more variably, different regions of the human leukocyte antigen (*HLA*) region.

## Treatment

The current treatment for KD is a high dose of 2 g/kg IVIG, given over 8–12 h [86]. The main goal of treatment is prevention of the development of CAA. Hypothesis on the mechanisms of efficacy of IVIG include immune modulation of T regulatory cells, neutralization of the etiologic agent, and reduction of cytokine production [8]. Treatment with IVIG significantly reduces the incidence of CAA [124]. IVIG is preferably given within the first 10 days after disease onset [83]. Apart from IVIG, high-dose aspirin is advised by the AHA, although evidence for further risk reduction for CAA is lacking [34, 124].

### Adjunctive use of treatment

The majority of patients respond rapidly to IVIG, yet approximately 10–20% of all patients do not respond or have recurrent fever within 36–48 h after IVIG. These children have a higher risk of developing CAA [53]. In Japan, risk-scores have been developed to identify patients with a higher risk of IVIG resistance [35, 66, 106]. Unfortunately, these risk-scores do not perform adequately in Western, ethnically mixed, and in Chinese populations [24, 75, 104, 112, 115]. A possible method to decrease IVIG resistance is to intensify the initial treatment. A recent meta-analysis showed a beneficial effect of adding corticosteroids to the initial treatment with IVIG, yet this effect was only found in Japanese studies and not in two studies conducted in the USA [16]. Burns et al. showed that adding infliximab to initial IVIG treatment did not decrease treatment resistance but did decrease the number of days with fever and inflammation parameters 24 h after treatment [125].

### Rescue treatment following IVIG non-response

Children who do not respond to IVIG require additional anti-inflammatory treatment. A second dose of IVIG is commonly advised, particularly in patients who have partially responded. Furthermore, corticosteroids are still commonly advised. The above-mentioned meta-analyses showed no significant benefit for either of these approaches when used as rescue treatment [16]. In 2012, we published the first case report successfully using the IL-1 antagonist anakinra for refractory KD [22]. Since then, additional clinical trials have been instigated to investigate both efficacy and safety of this IL-1 inhibitor [9]. Other secondary treatment possibilities are infliximab (TNF-α inhibitor), cyclosporine (calcineurin inhibitor), and statins, yet efficacy remains to be investigated [10].

### Additional treatment

After normalization of temperature, the AHA advises ongoing aspirin in a low dose until no evidence of CA dilation are present at by 4 to 6 weeks after the acute illness [83]. If CAA are present and persisting around that time, aspirin is continued as anti-thrombotic therapy. In case of large (around a *z*-score ≥10) or complex abnormalities, additional anticoagulation therapy should be administered to prevent clotting due to turbulence in these pro-coagulatory large coronary artery lesions [83, 87].

## Coronary artery aneurysms

Multiple criteria have been used for diagnosis of CAA. The criteria of the Japanese Circulation Society (JSC) state that an aneurysm is an artery of >3 mm in a child under the age of 5 and an artery of >4 mm in a child ≥5 years or when an arterial segment is 1.5 times its adjacent segment [31]. A giant CAA is classified as ≥8 mm or >4 times its adjacent segment [42]. Conversely, over the past years, it has become clear that *z*-scores, diameters adjusted for basal-surface-area, may be better indication of abnormality [25]. Multiple *z*-score classifications exist [23, 79, 82, 90]. Unfortunately, the *z-*scores using different classifications can vary, mainly at larger dimensions [101]. The threshold for abnormality is a *z*-score ≥2.5, although a *z*-score between 2 and 2.5 can be classified as a dilation [83]. A small-sized CAA has a *z*-score of 2.5–5, a medium-sized CAA of 5–10, and a giant CAA of ≥10 [79].

Risk factors for CAA have been inconsistently reported but include a male gender, a young age (<1 year), an incomplete disease presentation, IVIG resistance, and the duration of fever [43, 64, 113, 114].

### Imaging of CAA

**Fig. 2:**
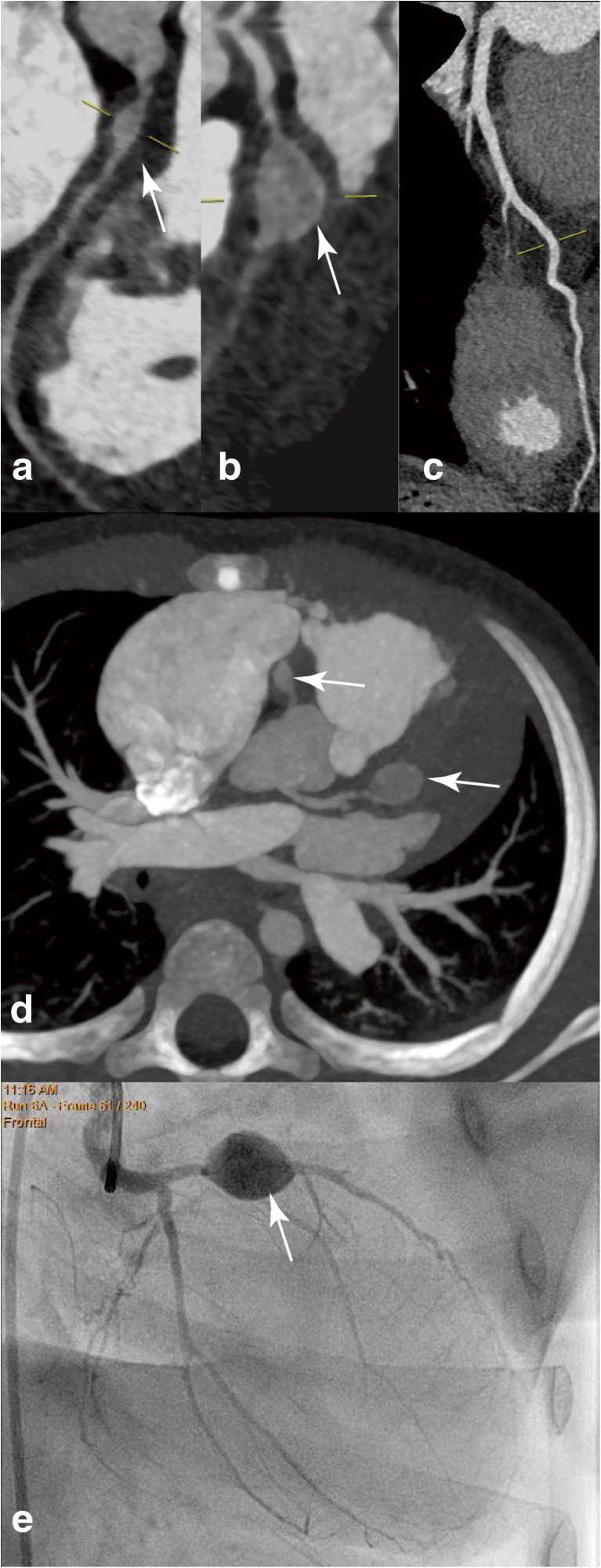
Imaging techniques used for Kawasaki disease. **a**, **b**, **d**, **e** display coronaries of the same patient with different imaging techniques. **a** Curved multi-planar reformat of the coronary computed tomography angiography (cCTA) scan shows an aneurysm of the right coronary artery. **b** A giant aneurysm of the left anterior descending artery. **c** A normal left anterior descending artery. **d** Thin slab maximum intensity projection of the aneurysmatic proximal right coronary artery and left anterior descending artery. **e** A clearly depicted giant aneurysm of the left anterior descending artery, visualized with coronary angiography

Multiple imaging techniques exist for the follow-up of patients after KD and in particular children with CAA. Echocardiography is a non-invasive method to image the coronary arteries, used in the acute phase of KD as well as during follow-up. With this non-invasive method, it is possible to evaluate the anatomy of the coronary arteries, myocardial function, and valve abnormalities. Nevertheless, it is impossible to visualize the distal coronary arteries with echocardiography. The gold standard for coronary anatomy is a conventional angiography, though this technique is invasive and exposes the patient to radiation. The role of cardiac MRI (cMRI) has been established over the past years [81, 122]. Using this modality, evaluation of the anatomy as well as cardiac function is possible. The disadvantage of cMRI is the need for anesthesia in younger children. CT angiography is an alternative. Although conventional CT angiography carries a high burden of radiation exposure, the new low-radiation dose CT scanning machines are becoming more widely available, which decreases the radiation burden significantly showing good resolution capacity in a small study [33]. In Fig. 2, a MRI and CT image of a normal and a giant CAA are depicted.

**Fig. 3:**
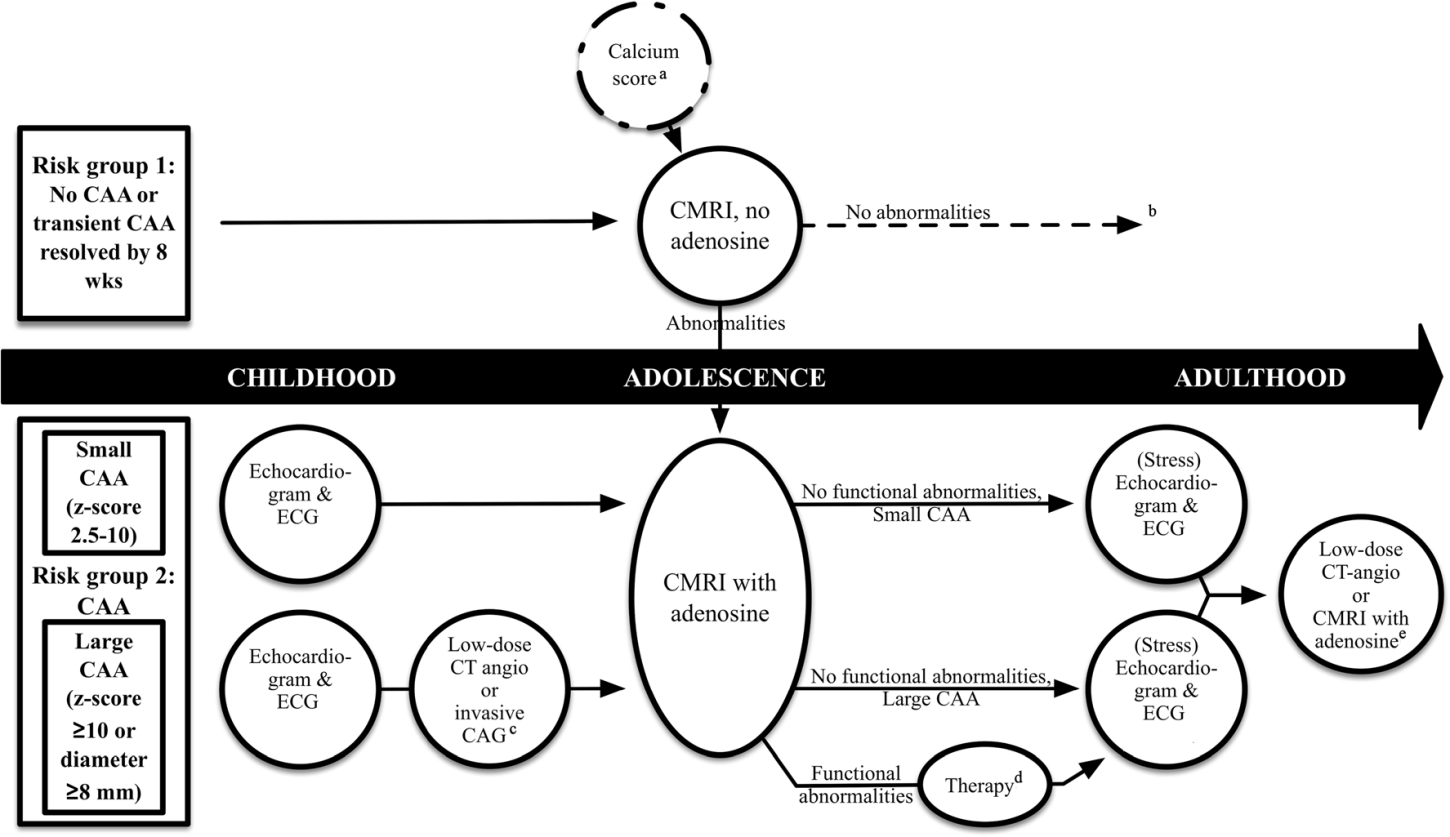
Flow diagram of the monitoring of Kawasaki disease using different imaging modalities. Footnote*:* Originally published in: Insights into imaging: Dietz SM, Tacke CEA, Kuipers IM, Wiegman A, de Winter RJ, Burns RC, Gordon RB, Groenink M, Kuijpers TW, Cardiovascular imaging in children and adults following Kawasaki disease, Insights into Imaging, 2015;6:697 (adapted version). ^a^When information is lacking about coronary arterial aneurysms (CAA) status, calcium score may be indicated as a screening method. If positive, a CMRI with adenosine should be performed. ^b^Long-term follow-up (cardiovascular counseling) of risk group 1 may be dictated by national health care policies and future studies. ^c^According to the availability and experience of a center with (low-dose) CT angiography. ^d^Which of the different revascularization options improves prognosis best is unclear to date. ^e^Additional tests to evaluate for progression to stenotic lesions

The AHA and JCS have both published guidelines on the follow-up of patients after KD [42, 83]. Recently, we proposed a pathway to follow-up patients after KD based on the worst-ever *z*-score [28]. This pathway includes a cMRI (with or without adenosine stress testing according to the CAA status) during adolescence for all patients to visualize the complete coronary tree and a more intensive follow-up for patients with CAA (Fig. 3).

### Natural history of CAA

Many CAA regress to a normal-sized lumen, mainly within the first 5 years. The likelihood of regression seems to be highly dependent on the original CAA size [19, 38]. While the lumen diameter may return to normal, it has become apparent that the vascular wall is often still damaged. Studies have shown persistent impaired dilation upon increased cardiac demand [39]. Also, changes in the coronary artery wall structure are seen, such as intimal hyperplasia [30, 54]. The remodeling of the coronary wall can cause potentially life-threatening situations as was shown by a case series of three adult patients who were released from follow-up care after normalization of the lumen but presented with chronic heart failure, unstable angina, and acute myocardial infarction [41].

### Consequences of CAA

(Giant) CAA may have serious long-term consequences. Apart from thrombosis within the CAA and perfusion abnormalities after the CAA, there is an increased risk of stenosis just proximal or distal to the CAA [59].

In a large study of 245 Japanese patients with giant CAA (≥8 mm), Tsuda et al. found 10-, 20-, and 30-year event-free survival rates of 64, 48, and 36%, respectively [127]. Fifteen patients died during follow-up. In another study of 76 Asian patients with giant CAA (≥8 mm), 10-, 20-, and 30-year survival rates of 95, 88, and 88% were reported [116]. In a recent study by Friedman et al., 21 major adverse cardiac events (MACE) took place in 90 patients with giant CAA at diagnosis (*z*-score ≥10) [38]. This indicates that patients with giant CAA are at considerable risk for MACE and risk continues to exist years after the acute phase of KD.

The risk for patients with small-medium-sized CAA is less clear. In a study by Chih et al., only 1/51 patients with medium-sized CAA (>4–8 mm) experienced ischemia during a median follow-up time of 47 months, although an additional 8 patients had stenosis and 4 patients developed calcification [19]. No patients with small-sized (localized dilatation with ≤4 mm diameter) CAA experienced MI. In the study by Friedman et al., no patients with small- (*z*-score 2.5–5) or medium-sized CAA (z-score 5–10) experienced any MACE. However, longer term studies will be needed to establish the long-term event rate in patients with small- or medium-sized CAA.

In patients with persisting CAA, calcifications are likely to develop as shown by a recent study looking at CT calcium scoring [56]. The data suggested this only occurs from approximately 10 years onwards following KD.

If thrombosis, stenosis, or abnormal blood flow in the CAA leads to a cardiac event or signs of ischemia, KD patients may need cardiac intervention. Thrombolytic treatment has been reported to be effective in treating fresh thrombus within giant CAA and in emergency management of ischemia due to thrombus [47].

Percutaneous coronary intervention (PCI) or coronary artery bypass grafting (CABG) can be used to revascularize the artery when stenotic lesions are present. CABG is used more often in multi-vessel disease. Percutaneous transluminal angioplasty may be complicated by the need of high balloon pressures in patients who have already developed calcification [1]. Two studies evaluating the difference between these two methods found that reinterventions were significantly higher in the PCI group [29, 84].

Available data are scarce on risk during pregnancy for women after KD. In a case series including 10 women who delivered 21 babies, none of the women experienced cardiovascular complications during pregnancy, including 4 women with CAA, MI, and CABG in the past [40]. In a following comprehensive review, 56 women with 81 deliveries were described and cardiovascular complications were reported in 7 cases, including 2 MIs during pregnancy [40].

Longer term studies will be needed to define the long term risk of CAA. However, the significant proportion of patients developing thrombosis within giant CAA, or suffering ischemic events due to stenosis, suggests that all patients with significant aneurysms following KD need life time follow-up and are at risk of cardiac complications long term.

## General cardiovascular risk

Apart from the increased cardiovascular risk due to persisting or regressed CAA, it is uncertain whether the vasculitis itself causes an increased cardiovascular or atherosclerotic risk at a later age. As most patients have not been followed long enough to evaluate the long-term natural course of the disease, multiple studies have focused on the use of surrogate markers for cardiovascular disease such as flow-mediated dilation, stiffness index, and carotid intima-media thickness (cIMT) [17, 89, 107]. Two reviews showed that most of these studies are small, lacking quality and there was significantly heterogeneity between studies [15, 27]. Nevertheless, results suggested that surrogate markers were increased in CAA-positive but not in CAA-negative patients. In a follow-up study evaluating cIMT, we found that patients with giant CAA have a trend towards an increased cIMT at a later age, whereas in children without any coronary artery enlargement, their cIMT was initially increased but normalized to control values over time [26]. The results suggest that long-term effects of KD are not caused by atherosclerosis, as one would expect the differences in cIMT to increase compared with control measurements. This is in concordance with a postmortem study in which multiple growth factors were seen in the smooth muscle cells and intima layer of the coronary arteries, but no fatty streaks as seen atherosclerosis, distinguishing “KD vasculopathy” from atherosclerosis [118].

## Quality of life and behavior

Multiple studies have investigated the cognitive and behavioral outcome after KD. Baker et al. studied 110 KD children, and found similar psychosocial and physical summary scores as an US population sample using a parent-completed questionnaire [3]. Only patients with giant CAA had a lower mean physical score. Parents did however report lower health perception. King et al. studied 38 KD patients and found no effect on cognitive or academic performance, but parents rated their children as having more internalizing and attentional behavior problems than controls [65]. Carlton-Conway et al. found that 40% of their patients showed internalizing scores in the clinical range as reported by parents, which was significantly more than their hospital controls who stayed in the hospital for a short period and had undergone cardiac catheterization [11]. Nishad et al. found no difference in social adaption, cognitive function, and behavioral function in 20 children [88]. Muta et al. studied 250 adolescents and young adults, including 19 patients with giant CAA and found significantly higher health-related quality of life (HRQOL) scores compared to national norms [85]. Two studies from our center showed significantly lower scores on several HRQOL scales in children under 5, when reported by their parents. However, self-report by the older KD children did not show any significant difference with controls. Moreover, parental perceptions of child vulnerability were significantly increased when compared to reference groups of Dutch parents [121, 129].

## Conclusion

Although many aspects of KD are still unknown, there is increasing knowledge on the origin and treatment of KD as well as the development and classification of CAA. Since children with previous KD are entering adulthood, long-term follow-up, with appropriate imaging modalities and awareness of the long-term effects, is increasingly important.

## Abbreviations

ABCC4: ATP-binding cassette, subfamily C, member 4
AHA: American Heart Association
ANGPT: Angiopoietin
CAA: Coronary artery aneurysms
CABG: Coronary artery bypass grafting
CIMT: Carotid intima-media thickness
cMRI: Cardiac MRI
DC: Dendritic cells
GWAS: Genome-wide association study
HRQOL: Health-related quality of life
ITPKC: Inositol-triphosphate 3-kinase
IVIG: Intravenous immunoglobulins
JSC: Japanese Circulation Society
KD: Kawasaki disease
NFAT: Nuclear factor of activated T cells
PCA: Percutaneous coronary intervention
SNP: Single nucleotide polymorphism
SPE: Streptococcal pyrogenic exotoxin
TGF-β: Transcription growth factor beta
TIE-2: Angiopoietin receptor
TSST-1: Toxic shock syndrome toxin-1
VEGF: Vascular endothelial growth factor

## References

[1] Akagi T (2005). Interventions in Kawasaki disease. Pediatr Cardiol.

[2] Alphonse MP, Duong TT, Shumitzu C, Hoang TL, McCrindle BW, Franco A, Schurmans S, Philpott DJ, Hibberd ML, Burns J, Kuijpers TW, Yeung RS (2016). Inositol-triphosphate 3-kinase C mediates inflammasome activation and treatment response in Kawasaki disease. J Immunol.

[3] Baker AL, Gauvreau K, Newburger JW, Sundel RP, Fulton DR, Jenkins KJ (2003). Physical and psychosocial health in children who have had Kawasaki disease. Pediatrics.

[4] Breunis WB, Biezeveld MH, Geissler J, Ottenkamp J, Kuipers IM, Lam J, Hutchinson A, Welch R, Chanock SJ, Kuijpers TW (2006). Vascular endothelial growth factor gene haplotypes in Kawasaki disease. Arthritis Rheum.

[5] Breunis WB, Davila S, Shimizu C, Oharaseki T, Takahashi K, van Houdt M, Khor CC, Wright VJ, Levin M, Burns JC, Burgner D, Hibberd ML, Kuijpers TW, International Kawasaki Disease Genetics C (2012). Disruption of vascular homeostasis in patients with Kawasaki disease: involvement of vascular endothelial growth factor and angiopoietins. Arthritis Rheum.

[6] Brown TJ, Crawford SE, Cornwall ML, Garcia F, Shulman ST, Rowley AH (2001). CD8 T lymphocytes and macrophages infiltrate coronary artery aneurysms in acute Kawasaki disease. J Infect Dis.

[7] Burgner D, Davila S, Breunis WB, Ng SB, Li Y, Bonnard C, Ling L, Wright VJ, Thalamuthu A, Odam M, Shimizu C, Burns JC, Levin M, Kuijpers TW, Hibberd ML, International Kawasaki Disease Genetics C (2009). A genome-wide association study identifies novel and functionally related susceptibility loci for Kawasaki disease. PLoS Genet.

[8] Burns JC, Franco A (2015). The immunomodulatory effects of intravenous immunoglobulin therapy in Kawasaki disease. Expert Rev Clin Immunol.

[9] Burns JC, Kone-Paut I, Kuijpers T, Shimizu C, Tremoulet A, Arditi M (2017). Review: found in translation: international initiatives pursuing interleukin-1 blockade for treatment of acute Kawasaki disease. Arthritis & rheumatology.

[10] Campbell AJ, Burns JC (2016). Adjunctive therapies for Kawasaki disease. J Inf Secur.

[11] Carlton-Conway D, Ahluwalia R, Henry L, Michie C, Wood L, Tulloh R (2005). Behaviour sequelae following acute Kawasaki disease. BMC Pediatr.

[12] Chang CJ, Kuo HC, Chang JS, Lee JK, Tsai FJ, Khor CC, Chang LC (2013). Replication and meta-analysis of GWAS identified susceptibility loci in Kawasaki disease confirm the importance of B lymphoid tyrosine kinase (BLK) in disease susceptibility. PLoS One.

[13] Chang LY, Lu CY, Shao PL, Lee PI, Lin MT, Fan TY, Cheng AL, Lee WL, Hu JJ, Yeh SJ, Chang CC, Chiang BL, Wu MH, Huang LM (2014). Viral infections associated with Kawasaki disease. J Formos Med Assoc.

[14] Chen JJ, Ma XJ, Liu F, Yan WL, Huang MR, Huang M, Huang GY, Shanghai Kawasaki Disease Research G (2016). Epidemiologic features of Kawasaki disease in Shanghai from 2008 through 2012. Pediatr Infect Dis J.

[15] Chen KY, Curtis N, Dahdah N, Kowalski R, Cheung M, Burgner DP (2016). Kawasaki disease and cardiovascular risk: a comprehensive review of subclinical vascular changes in the longer term. Acta Paediatr.

[16] Chen S, Dong Y, Kiuchi MG, Wang J, Li R, Ling Z, Zhou T, Wang Z, Martinek M, Purerfellner H, Liu S, Krucoff MW (2016). Coronary artery complication in Kawasaki disease and the importance of early intervention : a systematic review and meta-analysis. JAMA Pediatr.

[17] Cheung YF, O K, Woo CW, Armstrong S, Siow YL, Chow PC, Cheung EW (2008). Oxidative stress in children late after Kawasaki disease: relationship with carotid atherosclerosis and stiffness. BMC Pediatr.

[18] Chi H, Huang FY, Chen MR, Chiu NC, Lee HC, Lin SP, Chen WF, Lin CL, Chan HW, Liu HF, Huang LM, Lee YJ (2010). ITPKC gene SNP rs28493229 and Kawasaki disease in Taiwanese children. Hum Mol Genet.

[19] Chih WL, Wu PY, Sun LC, Lin MT, Wang JK, Wu MH (2016). Progressive coronary dilatation predicts worse outcome in Kawasaki disease. J Pediatr.

[20] Cho JH, Han MY, Cha SH, Jung JH, Yoon KL (2014). Genetic polymorphism of SMAD5 is associated with Kawasaki disease. Pediatr Cardiol.

[21] Choi YM, Shim KS, Yoon KL, Han MY, Cha SH, Kim SK, Jung JH (2012). Transforming growth factor beta receptor II polymorphisms are associated with Kawasaki disease. Korean J Pediatr.

[22] Cohen S, Tacke CE, Straver B, Meijer N, Kuipers IM, Kuijpers TW (2012). A child with severe relapsing Kawasaki disease rescued by IL-1 receptor blockade and extracorporeal membrane oxygenation. Ann Rheum Dis.

[23] Dallaire F, Dahdah N (2011). New equations and a critical appraisal of coronary artery Z scores in healthy children. J Am Soc Echocardiogr.

[24] Davies S, Sutton N, Blackstock S, Gormley S, Hoggart CJ, Levin M, Herberg JA (2015). Predicting IVIG resistance in UK Kawasaki disease. Arch Dis Child.

[25] de Zorzi A, Colan SD, Gauvreau K, Baker AL, Sundel RP, Newburger JW (1998). Coronary artery dimensions may be misclassified as normal in Kawasaki disease. J Pediatr.

[26] Dietz SM, Tacke CE, de Groot E, Kuipers IM, Hutten BA, Kuijpers TW, Group DKS (2016). Extracardial vasculopathy after Kawasaki disease: a long-term follow-up study. J Am Heart Assoc.

[27] Dietz SM, Tacke CE, Hutten BA, Kuijpers TW (2015). Peripheral endothelial (dys)function, arterial stiffness and carotid intima-media thickness in patients after Kawasaki disease: a systematic review and meta-analyses. PLoS One.

[28] Dietz SM, Tacke CE, Kuipers IM, Wiegman A, de Winter RJ, Burns JC, Gordon JB, Groenink M, Kuijpers TW (2015). Cardiovascular imaging in children and adults following Kawasaki disease. Insights Imaging.

[29] Dionne A, Bakloul M, Manlhiot C, McCrindle BW, Hosking M, Houde C, Pepelassis D, Dahdah N (2017). Coronary artery bypass grafting and percutaneous coronary intervention after Kawasaki disease: the pediatric Canadian series. Pediatr Cardiol.

[30] Dionne A, Ibrahim R, Gebhard C, Bakloul M, Selly JB, Leye M, Dery J, Lapierre C, Girard P, Fournier A, Dahdah N (2015). Coronary wall structural changes in patients with Kawasaki disease: new insights from optical coherence tomography (OCT). J Am Heart Assoc.

[31] Research committee on Kawasaki Disease (1984) Report of subcommittee on standardization of diagnostic criteria and reporting of coronary artery lesions in Kawasaki disease. Ministry of Health and Welfare, Tokyo

[32] Duan J, Lou J, Zhang Q, Ke J, Qi Y, Shen N, Zhu B, Zhong R, Wang Z, Liu L, Wu J, Wang W, Gong F, Miao X (2014). A genetic variant rs1801274 in FCGR2A as a potential risk marker for Kawasaki disease: a case-control study and meta-analysis. PLoS One.

[33] Duan Y, Wang X, Cheng Z, Wu D, Wu L (2012). Application of prospective ECG-triggered dual-source CT coronary angiography for infants and children with coronary artery aneurysms due to Kawasaki disease. Br J Radiol.

[34] Durongpisitkul K, Gururaj VJ, Park JM, Martin CF (1995). The prevention of coronary artery aneurysm in Kawasaki disease: a meta-analysis on the efficacy of aspirin and immunoglobulin treatment. Pediatrics.

[35] Egami K, Muta H, Ishii M, Suda K, Sugahara Y, Iemura M, Matsuishi T (2006). Prediction of resistance to intravenous immunoglobulin treatment in patients with Kawasaki disease. J Pediatr.

[36] Fraison JB, Seve P, Dauphin C, Mahr A, Gomard-Mennesson E, Varron L, Pugnet G (2016). Kawasaki disease in adults: observations in France and literature review. Autoimmun Rev.

[37] Franco A, Touma R, Song Y, Shimizu C, Tremoulet AH, Kanegaye JT, Burns JC (2014). Specificity of regulatory T cells that modulate vascular inflammation. Autoimmunity.

[38] Friedman KG, Gauvreau K, Hamaoka-Okamoto A, Tang A, Berry E, Tremoulet AH, Mahavadi VS, Baker A, deFerranti SD, Fulton DR, Burns JC, Newburger JW (2016). Coronary artery aneurysms in Kawasaki disease: risk factors for progressive disease and adverse cardiac events in the US population. J Am Heart Assoc.

[39] Furuyama H, Odagawa Y, Katoh C, Iwado Y, Ito Y, Noriyasu K, Mabuchi M, Yoshinaga K, Kuge Y, Kobayashi K, Tamaki N (2003). Altered myocardial flow reserve and endothelial function late after Kawasaki disease. J Pediatr.

[40] Gordon CT, Jimenez-Fernandez S, Daniels LB, Kahn AM, Tarsa M, Matsubara T, Shimizu C, Burns JC, Gordon JB (2014). Pregnancy in women with a history of Kawasaki disease: management and outcomes. BJOG.

[41] Gordon JB, Daniels LB, Kahn AM, Jimenez-Fernandez S, Vejar M, Numano F, Burns JC (2016). The spectrum of cardiovascular lesions requiring intervention in adults after Kawasaki disease. JACC Cardiovasc Interv.

[42] Group JCSJW (2014). Guidelines for diagnosis and management of cardiovascular sequelae in Kawasaki disease (JCS 2013). Digest version. Circ J: Off J Jpn Circ Soc.

[43] Ha KS, Jang G, Lee J, Lee K, Hong Y, Son C, Lee J (2013). Incomplete clinical manifestation as a risk factor for coronary artery abnormalities in Kawasaki disease: a meta-analysis. Eur J Pediatr.

[44] Ha S, Seo GH, Kim KY, Kim DS (2016). Epidemiologic study on Kawasaki disease in Korea, 2007-2014: based on health insurance review & assessment service claims. J Korean Med Sci.

[45] Hall GC, Tulloh LE, Tulloh RM (2016). Kawasaki disease incidence in children and adolescents: an observational study in primary care. Br J Gen Pract.

[46] Hao S, Jin B, Tan Z, Li Z, Ji J, Hu G, Wang Y, Deng X, Kanegaye JT, Tremoulet AH, Burns JC, Cohen HJ, Ling XB, Pediatric Emergency Medicine Kawasaki Disease Research G (2016). A classification tool for differentiation of Kawasaki disease from other febrile illnesses. J Pediatr.

[47] Harada M, Akimoto K, Ogawa S, Kato H, Nakamura Y, Hamaoka K, Saji T, Shimizu T, Kato T (2013). National Japanese survey of thrombolytic therapy selection for coronary aneurysm: intracoronary thrombolysis or intravenous coronary thrombolysis in patients with Kawasaki disease. Pediatr Int.

[48] Harnick DJ, Jayaraman T, Ma Y, Mulieri P, Go LO, Marks AR (1995). The human type 1 inositol 1,4,5-trisphosphate receptor from T lymphocytes. Structure, localization, and tyrosine phosphorylation. J Biol Chem.

[49] Henn V, Slupsky JR, Grafe M, Anagnostopoulos I, Forster R, Muller-Berghaus G, Kroczek RA (1998). CD40 ligand on activated platelets triggers an inflammatory reaction of endothelial cells. Nature.

[50] Hoang LT, Shimizu C, Ling L, Naim AN, Khor CC, Tremoulet AH, Wright V, Levin M, Hibberd ML, Burns JC (2014). Global gene expression profiling identifies new therapeutic targets in acute Kawasaki disease. Genome Med.

[51] Hsueh KC, Lin YJ, Chang JS, Wan L, Tsai YH, Tsai CH, Chen CP, Tsai FJ (2008). Association of vascular endothelial growth factor C-634 g polymorphism in Taiwanese children with Kawasaki disease. Pediatr Cardiol.

[52] Hui-Yuen JS, Duong TT, Yeung RS (2006). TNF-alpha is necessary for induction of coronary artery inflammation and aneurysm formation in an animal model of Kawasaki disease. J Immunol.

[53] Hwang JY, Lee KY, Rhim JW, Youn YS, Oh JH, Han JW, Lee JS, Burgner D (2011). Assessment of intravenous immunoglobulin non-responders in Kawasaki disease. Arch Dis Child.

[54] Iemura M, Ishii M, Sugimura T, Akagi T, Kato H (2000). Long term consequences of regressed coronary aneurysms after Kawasaki disease: vascular wall morphology and function. Heart.

[55] Jakob A, Whelan J, Kordecki M, Berner R, Stiller B, Arnold R, von Kries R, Neumann E, Roubinis N, Robert M, Grohmann J, Hohn R, Hufnagel M (2016). Kawasaki disease in Germany: a prospective, population-based study adjusted for underreporting. Pediatr Infect Dis J.

[56] Kahn AM, Budoff MJ, Daniels LB, Jimenez-Fernandez S, Cox AS, Gordon JB, Burns JC (2012). Calcium scoring in patients with a history of Kawasaki disease. JACC Cardiovasc Imaging.

[57] Kao AS, Getis A, Brodine S, Burns JC (2008). Spatial and temporal clustering of Kawasaki syndrome cases. Pediatr Infect Dis J.

[58] Kariyazono H, Ohno T, Khajoee V, Ihara K, Kusuhara K, Kinukawa N, Mizuno Y, Hara T (2004). Association of vascular endothelial growth factor (VEGF) and VEGF receptor gene polymorphisms with coronary artery lesions of Kawasaki disease. Pediatr Res.

[59] Kato H, Sugimura T, Akagi T, Sato N, Hashino K, Maeno Y, Kazue T, Eto G, Yamakawa R (1996). Long-term consequences of Kawasaki disease. A 10- to 21-year follow-up study of 594 patients. Circulation.

[60] Kawasaki T (1967). Acute febrile mucocutaneous syndrome with lymphoid involvement with specific desquamation of the fingers and toes in children. Arerugi.

[61] Khor CC, Davila S, Breunis WB, Lee YC, Shimizu C, Wright VJ, Yeung RS (2011). Genome-wide association study identifies FCGR2A as a susceptibility locus for Kawasaki disease. Nat Genet.

[62] Khor CC, Davila S, Shimizu C, Sheng S, Matsubara T, Suzuki Y, Newburger JW, Baker A, Burgner D, Breunis W, Kuijpers T, Wright VJ, Levin M, Hibberd ML, Burns JC, US, International Kawasaki Disease Genetics C (2011). Genome-wide linkage and association mapping identify susceptibility alleles in ABCC4 for Kawasaki disease. J Med Genet.

[63] Kim JJ, Hong YM, Sohn S, Jang GY, Ha KS, Yun SW, Han MK, Lee KY, Song MS, Lee HD, Kim DS, Lee JE, Shin ES, Jang JH, Lee YS, Kim SY, Lee JY, Han BG, Wu JY, Kim KJ, Park YM, Seo EJ, Park IS, Lee JK, Korean Kawasaki Disease Genetics C (2011). A genome-wide association analysis reveals 1p31 and 2p13.3 as susceptibility loci for Kawasaki disease. Hum Genet.

[64] Kim T, Choi W, Woo CW, Choi B, Lee J, Lee K, Son C, Lee J (2007). Predictive risk factors for coronary artery abnormalities in Kawasaki disease. Eur J Pediatr.

[65] King WJ, Schlieper A, Birdi N, Cappelli M, Korneluk Y, Rowe PC (2000). The effect of Kawasaki disease on cognition and behavior. Arch Pediatr Adolesc Med.

[66] Kobayashi T, Inoue Y, Takeuchi K, Okada Y, Tamura K, Tomomasa T, Kobayashi T, Morikawa A (2006). Prediction of intravenous immunoglobulin unresponsiveness in patients with Kawasaki disease. Circulation.

[67] Kuo HC, Chao MC, Hsu YW, Lin YC, Huang YH, Yu HR, Hou MF, Liang CD, Yang KD, Chang WC, Wang CL (2012). CD40 Gene polymorphisms associated with susceptibility and coronary artery lesions of Kawasaki disease in the Taiwanese population. ScientificWorldJournal.

[68] Kuo HC, Hsu YW, Lo MH, Huang YH, Chien SC, Chang WC (2014). Single-nucleotide polymorphism rs7251246 in ITPKC is associated with susceptibility and coronary artery lesions in Kawasaki disease. PLoS One.

[69] Kuo HC, Li SC, Guo MM, Huang YH, Yu HR, Huang FC, Jiao F, Kuo HC, Andrade J, Chan WC (2016). Genome-wide association study identifies novel susceptibility genes associated with coronary artery aneurysm formation in Kawasaki disease. PLoS One.

[70] Kuo HC, Onouchi Y, Hsu YW, Chen WC, Huang JD, Huang YH, Yang YL, Chao MC, Yu HR, Juan YS, Kuo CM, Yang KD, Huang JS, Chang WC (2011). Polymorphisms of transforming growth factor-beta signaling pathway and Kawasaki disease in the Taiwanese population. J Hum Genet.

[71] Kuo HC, Yang KD, Juo SH, Liang CD, Chen WC, Wang YS, Lee CH, Hsi E, Yu HR, Woon PY, Lin IC, Huang CF, Hwang DY, Lee CP, Lin LY, Chang WP, Chang WC (2011). ITPKC single nucleotide polymorphism associated with the Kawasaki disease in a Taiwanese population. PLoS One.

[72] Lee YC, Kuo HC, Chang JS, Chang LY, Huang LM, Chen MR, Liang CD, Chi H, Huang FY, Lee ML, Huang YC, Hwang B, Chiu NC, Hwang KP, Lee PC, Chang LC, Liu YM, Chen YJ, Chen CH, Taiwan Pediatric IDA, Chen YT, Tsai FJ, Wu JY (2012). Two new susceptibility loci for Kawasaki disease identified through genome-wide association analysis. Nat Genet.

[73] Lin MT, Wang JK, Yeh JI, Sun LC, Chen PL, Wu JF, Chang CC, Lee WL, Shen CT, Wang NK, Wu CS, Yeh SZ, Chen CA, Chiu SN, Wu MH (2011). Clinical implication of the C allele of the ITPKC gene SNP rs28493229 in Kawasaki disease: association with disease susceptibility and BCG scar reactivation. Pediatr Infect Dis J.

[74] Ling XB, Kanegaye JT, Ji J, Peng S, Sato Y, Tremoulet A, Burns JC, Cohen HJ (2013). Point-of-care differentiation of Kawasaki disease from other febrile illnesses. J Pediatr.

[75] Loomba RS, Raskin A, Gudausky TM, Kirkpatrick E (2016). Role of the Egami score in predicting intravenous immunoglobulin resistance in Kawasaki disease among different ethnicities. Am J Ther.

[76] Lou J, Xu S, Zou L, Zhong R, Zhang T, Sun Y, Lu X, Liu L, Li C, Wang L, Xiong G, Wang W, Gong F, Wu J (2012). A functional polymorphism, rs28493229, in ITPKC and risk of Kawasaki disease: an integrated meta-analysis. Mol Biol Rep.

[77] Lou J, Zhong R, Shen N, Lu XZ, Ke JT, Duan JY, Qi YQ, Wang YJ, Zhang Q, Wang W, Gong FQ, Miao XP (2015). Systematic confirmation study of GWAS-identified genetic variants for Kawasaki disease in a Chinese population. Sci Rep.

[78] Makino N, Nakamura Y, Yashiro M, Ae R, Tsuboi S, Aoyama Y, Kojo T, Uehara R, Kotani K, Yanagawa H (2015). Descriptive epidemiology of Kawasaki disease in Japan, 2011-2012: from the results of the 22nd nationwide survey. J Epidemiol.

[79] Manlhiot C, Millar K, Golding F, McCrindle BW (2010). Improved classification of coronary artery abnormalities based only on coronary artery z-scores after Kawasaki disease. Pediatr Cardiol.

[80] Matsubara K, Fukaya T, Miwa K, Shibayama N, Nigami H, Harigaya H, Nozaki H, Hirata T, Baba K, Suzuki T, Ishiguro A (2006). Development of serum IgM antibodies against superantigens of *Staphylococcus aureus* and *Streptococcus pyogenes* in Kawasaki disease. Clin Exp Immunol.

[81] Mavrogeni S, Papadopoulos G, Douskou M, Kaklis S, Seimenis I, Baras P, Nikolaidou P, Bakoula C, Karanasios E, Manginas A, Cokkinos DV (2004). Magnetic resonance angiography is equivalent to X-ray coronary angiography for the evaluation of coronary arteries in Kawasaki disease. J Am Coll Cardiol.

[82] McCrindle BW, Li JS, Minich LL, Colan SD, Atz AM, Takahashi M, Vetter VL, Gersony WM, Mitchell PD, Newburger JW, Pediatric Heart Network I (2007). Coronary artery involvement in children with Kawasaki disease: risk factors from analysis of serial normalized measurements. Circulation.

[83] McCrindle BW, Rowley AH, Newburger JW, Burns JC, Bolger AF, Gewitz M, Baker AL, Jackson MA, Takahashi M, Shah PB, Kobayashi T, Wu MH, Saji TT, Pahl E, American Heart Association Rheumatic Fever E, Kawasaki Disease Committee of the Council on Cardiovascular Disease in the Y, Council on C, Stroke N, Council on Cardiovascular S, Anesthesia, Council on E, Prevention (2017) Diagnosis, treatment, and long-term management of kawasaki disease: a scientific statement for health professionals from the American Heart Association. Circulation 135:e927–e999 10.1161/CIR.0000000000000484 28356445

[84] Muta H, Ishii M (2010). Percutaneous coronary intervention versus coronary artery bypass grafting for stenotic lesions after Kawasaki disease. J Pediatr.

[85] Muta H, Ishii M, Iemura M, Matsuishi T (2010). Health-related quality of life in adolescents and young adults with a history of Kawasaki disease. J Pediatr.

[86] Newburger JW, Takahashi M, Beiser AS, Burns JC, Bastian J, Chung KJ, Colan SD, Duffy CE, Fulton DR, Glode MP (1991). A single intravenous infusion of gamma globulin as compared with four infusions in the treatment of acute Kawasaki syndrome. N Engl J Med.

[87] Newburger JW, Takahashi M, Burns JC (2016). Kawasaki disease. J Am Coll Cardiol.

[88] Nishad P, Singh S, Sidhu M, Malhi P (2010). Cognitive and behaviour assessment following Kawasaki disease—a study from North India. Rheumatol Int.

[89] Noto N, Okada T, Karasawa K, Ayusawa M, Sumitomo N, Harada K, Mugishima H (2009). Age-related acceleration of endothelial dysfunction and subclinical atherosclerosis in subjects with coronary artery lesions after Kawasaki disease. Pediatr Cardiol.

[90] Olivieri L, Arling B, Friberg M, Sable C (2009). Coronary artery Z score regression equations and calculators derived from a large heterogeneous population of children undergoing echocardiography. J Am Soc Echocardiogr.

[91] Onouchi Y (2012). Genetics of Kawasaki disease: what we know and don’t know. Circ J.

[92] Onouchi Y, Gunji T, Burns JC, Shimizu C, Newburger JW, Yashiro M, Nakamura Y (2008). ITPKC functional polymorphism associated with Kawasaki disease susceptibility and formation of coronary artery aneurysms. Nat Genet.

[93] Onouchi Y, Onoue S, Tamari M, Wakui K, Fukushima Y, Yashiro M, Nakamura Y, Yanagawa H, Kishi F, Ouchi K, Terai M, Hamamoto K, Kudo F, Aotsuka H, Sato Y, Nariai A, Kaburagi Y, Miura M, Saji T, Kawasaki T, Nakamura Y, Hata A (2004). CD40 ligand gene and Kawasaki disease. Eur J Hum Genet.

[94] Onouchi Y, Ozaki K, Burns JC, Shimizu C, Terai M, Hamada H, Honda T (2012). A genome-wide association study identifies three new risk loci for Kawasaki disease. Nat Genet.

[95] Onouchi Y, Suzuki Y, Suzuki H, Terai M, Yasukawa K, Hamada H, Suenaga T (2013). ITPKC and CASP3 polymorphisms and risks for IVIG unresponsiveness and coronary artery lesion formation in Kawasaki disease. Pharmacogenomics J.

[96] Parthasarathy P, Agarwal A, Chawla K, Tofighi T, Mondal TK (2015). Upcoming biomarkers for the diagnosis of Kawasaki disease: a review. Clin Biochem.

[97] Peng Q, Chen C, Zhang Y, He H, Wu Q, Liao J, Li B, Luo C, Hu X, Zheng Z, Yang Y (2012). Single-nucleotide polymorphism rs2290692 in the 3′UTR of ITPKC associated with susceptibility to Kawasaki disease in a Han Chinese population. Pediatr Cardiol.

[98] Peng Q, Deng Y, Yang X, Leng X, Yang Y, Liu H (2016). Genetic variants of ADAM17 are implicated in the pathological process of Kawasaki disease and secondary coronary artery lesions via the TGF-beta/SMAD3 signaling pathway. Eur J Pediatr.

[99] Phuong LK, Bonetto C, Buttery J, Pernus YB, Chandler R, Goldenthal KL, Kucuku M, Monaco G, Pahud B, Shulman ST, Top KA, Ulloa-Gutierrez R, Varricchio F, de Ferranti S, Newburger JW, Dahdah N, Singh S, Bonhoeffer J, Burgner D, Brighton Collaboration Kawasaki Disease Working G (2016). Kawasaki disease and immunisation: standardised case definition & guidelines for data collection, analysis. Vaccine.

[100] Reth M, Wienands J (1997). Initiation and processing of signals from the B cell antigen receptor. Annu Rev Immunol.

[101] Ronai C, Hamaoka-Okamoto A, Baker AL, de Ferranti SD, Colan SD, Newburger JW, Friedman KG (2016). Coronary artery aneurysm measurement and Z score variability in Kawasaki disease. J Am Soc Echocardiogr: Off Publ Am Soc Echocardiogr.

[102] Rowley AH, Baker SC, Orenstein JM, Shulman ST (2008). Searching for the cause of Kawasaki disease—cytoplasmic inclusion bodies provide new insight. Nat Rev Microbiol.

[103] Rowley AH, Baker SC, Shulman ST, Rand KH, Tretiakova MS, Perlman EJ, Garcia FL, Tajuddin NF, Fox LM, Huang JH, Ralphe JC, Takahashi K, Flatow J, Lin S, Kalelkar MB, Soriano B, Orenstein JM (2011). Ultrastructural, immunofluorescence, and RNA evidence support the hypothesis of a “new” virus associated with Kawasaki disease. J Infect Dis.

[104] Sanchez-Manubens J, Anton J, Bou R, Iglesias E, Calzada-Hernandez J, Borlan S, Gimenez-Roca C, Rivera J, Kawasaki Disease in Catalonia Working G (2016). Role of the Egami score to predict immunoglobulin resistance in Kawasaki disease among a Western Mediterranean population. Rheumatol Int.

[105] Sanchez-Manubens J, Anton J, Bou R, Iglesias E, Calzada-Hernandez J, Kawasaki Disease in Catalonia Working G (2016). Incidence, epidemiology and clinical features of Kawasaki disease in Catalonia, Spain. Clin Exp Rheumatol.

[106] Sano T, Kurotobi S, Matsuzaki K, Yamamoto T, Maki I, Miki K, Kogaki S, Hara J (2007). Prediction of non-responsiveness to standard high-dose gamma-globulin therapy in patients with acute Kawasaki disease before starting initial treatment. Eur J Pediatr.

[107] Selamet Tierney ES, Gal D, Gauvreau K, Baker AL, Trevey S, O’Neill SR, Jaff MR, de Ferranti S, Fulton DR, Colan SD, Newburger JW (2013). Vascular health in Kawasaki disease. J Am Coll Cardiol.

[108] Shendre A, Wiener HW, Zhi D, Vazquez AI, Portman MA, Shrestha S (2014). High-density genotyping of immune loci in Kawasaki disease and IVIG treatment response in European-American case-parent trio study. Genes Immun.

[109] Shimizu C, Jain S, Davila S, Hibberd ML, Lin KO, Molkara D, Frazer JR (2011). Transforming growth factor-beta signaling pathway in patients with Kawasaki disease. Circ Cardiovasc Genet.

[110] Shrestha S, Wiener H, Shendre A, Kaslow RA, Wu J, Olson A, Bowles NE, Patel H, Edberg JC, Portman MA (2012). Role of activating FcgammaR gene polymorphisms in Kawasaki disease susceptibility and intravenous immunoglobulin response. Circ Cardiovasc Genet.

[111] Shrestha S, Wiener HW, Olson AK, Edberg JC, Bowles NE, Patel H, Portman MA (2011). Functional FCGR2B gene variants influence intravenous immunoglobulin response in patients with Kawasaki disease. J Allergy Clin Immunol.

[112] Sleeper LA, Minich LL, McCrindle BM, Li JS, Mason W, Colan SD, Atz AM, Printz BF, Baker A, Vetter VL, Newburger JW, Pediatric Heart Network I (2011). Evaluation of Kawasaki disease risk-scoring systems for intravenous immunoglobulin resistance. J Pediatr.

[113] Son MB, Gauvreau K, Ma L, Baker AL, Sundel RP, Fulton DR, Newburger JW (2009). Treatment of Kawasaki disease: analysis of 27 US pediatric hospitals from 2001 to 2006. Pediatrics.

[114] Song D, Yeo Y, Ha K, Jang G, Lee J, Lee K, Son C, Lee J (2009). Risk factors for Kawasaki disease-associated coronary abnormalities differ depending on age. Eur J Pediatr.

[115] Song R, Yao W, Li X (2016) Efficacy of four scoring systems in predicting intravenous immunoglobulin resistance in children with Kawasaki disease in a children’s hospital in Beijing, North China. J Pediatr 184:120–124 10.1016/j.jpeds.2016.12.018 28043682

[116] Suda K, Iemura M, Nishiono H, Teramachi Y, Koteda Y, Kishimoto S, Kudo Y, Itoh S, Ishii H, Ueno T, Tashiro T, Nobuyoshi M, Kato H, Matsuishi T (2011). Long-term prognosis of patients with Kawasaki disease complicated by giant coronary aneurysms: a single-institution experience. Circulation.

[117] Suenaga T, Suzuki H, Shibuta S, Takeuchi T, Yoshikawa N (2009). Detection of multiple superantigen genes in stools of patients with Kawasaki disease. J Pediatr.

[118] Suzuki A, Miyagawa-Tomita S, Komatsu K, Nishikawa T, Sakomura Y, Horie T, Nakazawa M (2000). Active remodeling of the coronary arterial lesions in the late phase of Kawasaki disease: immunohistochemical study. Circulation.

[119] Suzuki H, Uemura S, Tone S, Iizuka T, Koike M, Hirayama K, Maeda J (1996). Effects of immunoglobulin and gamma-interferon on the production of tumour necrosis factor-alpha and interleukin-1 beta by peripheral blood monocytes in the acute phase of Kawasaki disease. Eur J Pediatr.

[120] Tacke CE, Breunis WB, Pereira RR, Breur JM, Kuipers IM, Kuijpers TW (2014). Five years of Kawasaki disease in the Netherlands: a national surveillance study. Pediatr Infect Dis J.

[121] Tacke CE, Haverman L, Berk BM, van Rossum MA, Kuipers IM, Grootenhuis MA, Kuijpers TW (2012). Quality of life and behavioral functioning in Dutch children with a history of Kawasaki disease. J Pediatr.

[122] Tacke CE, Romeih S, Kuipers IM, Spijkerboer AM, Groenink M, Kuijpers TW (2013). Evaluation of cardiac function by magnetic resonance imaging during the follow-up of patients with Kawasaki disease. Circ Cardiovasc Imaging.

[123] Taniuchi S, Masuda M, Teraguchi M, Ikemoto Y, Komiyama Y, Takahashi H, Kino M, Kobayashi Y (2005). Polymorphism of fc gamma RIIa may affect the efficacy of gamma-globulin therapy in Kawasaki disease. J Clin Immunol.

[124] Terai M, Shulman ST (1997). Prevalence of coronary artery abnormalities in Kawasaki disease is highly dependent on gamma globulin dose but independent of salicylate dose. J Pediatr.

[125] Tremoulet AH, Jain S, Jaggi P, Jimenez-Fernandez S, Pancheri JM, Sun X, Kanegaye JT, Kovalchin JP, Printz BF, Ramilo O, Burns JC (2014). Infliximab for intensification of primary therapy for Kawasaki disease: a phase 3 randomised, double-blind, placebo-controlled trial. Lancet.

[126] Tsai FJ, Lee YC, Chang JS, Huang LM, Huang FY, Chiu NC, Chen MR, Chi H, Lee YJ, Chang LC, Liu YM, Wang HH, Chen CH, Chen YT, Wu JY (2011). Identification of novel susceptibility loci for Kawasaki disease in a Han Chinese population by a genome-wide association study. PLoS One.

[127] Tsuda E, Hamaoka K, Suzuki H, Sakazaki H, Murakami Y, Nakagawa M, Takasugi H, Yoshibayashi M (2014). A survey of the 3-decade outcome for patients with giant aneurysms caused by Kawasaki disease. Am Heart J.

[128] Turnier JL, Anderson MS, Heizer HR, Jone PN, Glode MP, Dominguez SR (2015). Concurrent respiratory viruses and Kawasaki disease. Pediatrics.

[129] van Oers HA, Tacke CE, Haverman L, Kuipers IM, Maurice-Stam H, Kuijpers TW, Grootenhuis MA (2014). Health related quality of life and perceptions of child vulnerability among parents of children with a history of Kawasaki disease. Acta Paediatr.

[130] Yan Y, Ma Y, Liu Y, Hu H, Shen Y, Zhang S, Ma Y, Tao D, Wu Q, Peng Q, Yang Y (2013). Combined analysis of genome-wide-linked susceptibility loci to Kawasaki disease in Han Chinese. Hum Genet.

[131] Yeung RS (2010). Kawasaki disease: update on pathogenesis. Curr Opin Rheumatol.

[132] Yoon KL (2015). Update of genetic susceptibility in patients with Kawasaki disease. Korean J Pediatr.

